# What calcium channels remember

**DOI:** 10.7554/eLife.90546

**Published:** 2023-08-11

**Authors:** Thomas Kaas, Jana Nerlich, Stefan Hallermann

**Affiliations:** 1 Carl-Ludwig-Institute of Physiology, Faculty of Medicine, Leipzig University Leipzig Germany

**Keywords:** synapse, axon, transmitter release, patch-clamp, Ca2+ channels, Rat

## Abstract

A new mechanism involving intermediate gating states of calcium channels explains how analogue postsynaptic potentials influence neurotransmitter release.

**Related research article** Trigo FF, Kawaguchi S. 2023. Analogue signaling of somato-dendritic synaptic activity to axon enhances GABA release in young cerebellar molecular layer interneurons. *eLife* **12**:e85971. doi: 10.7554/eLife.85971.

Computers are often compared to brains. Both receive input from the outside world, which is then analyzed and transformed into a reaction. Yet, information processing in our brains and computers is fundamentally different (Nagarajan and Stevens, 2008). Unlike computers, which solely rely on digital signals to process information (i.e., values can only be 1 or 0), neurons in human brains use a mix of analogue (signals of varying values) and digital coding to communicate effectively.

Neurons receive input from other neurons through their cell bodies and dendrites at one end and transmit information through wirelike axons at the other end. In the classical view, information transfer in the form of neurotransmitters on the dendritic end would fluctuate continuously (representing a continuous but varying analogue signal), until a voltage threshold would be reached that causes axons to fire electrical impulses (also known as an all-or-none action potentials). This would then lead to the release of neurotransmitters onto the next neuron. So, while signals in the dendrites are represented in an analog fashion, they were thought to be transmitted in a digital way along the axon (Figure 1A left).

**Figure 1.**
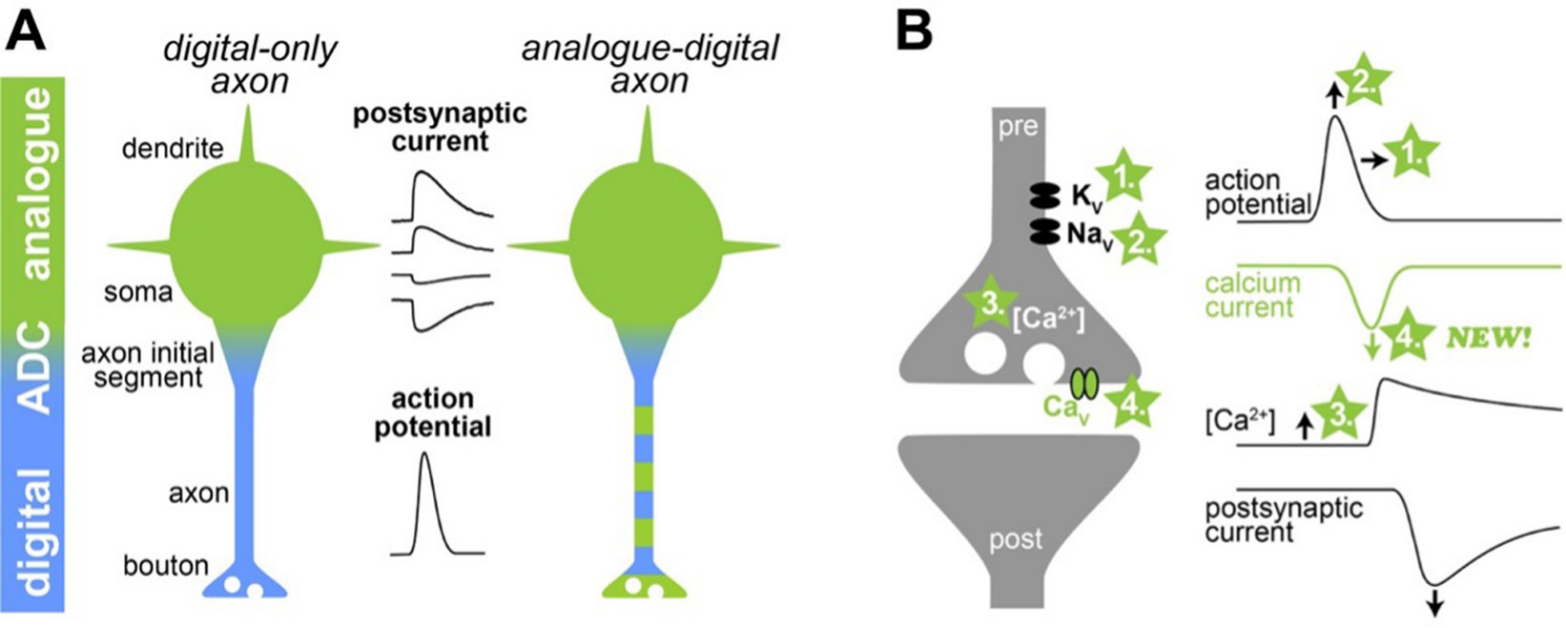
Illustration of a new mechanism of analogue-digital signaling. (**A**) *Left:* Neurons were thought to receive analogue input signals (green) in the form of gradual, postsynaptic potentials. These were then converted into digital output signals (blue) in the form of action potentials via an analogue-digital converter (ADC). *Right:* However, previous research has shown that axons can use a combination of analogue-digital signaling in the axon (green-blue dashed color). (**B**) *Left:* Schematic illustration of a synapse (pre and post synapse in grey) with voltage-dependent potassium, sodium and calcium channels (K_v_, Na_v_, and Ca_v_, respectively). Four mechanisms mediating analogue-digital signaling are indicated. *Right:* Presynaptic action potential, calcium current, residual calcium concentration, and the postsynaptic current are shown versus time (modified from Geiger and Jonas, 2000). In the proposed fourth mechanism by Trigo and Kawaguchi, analogue signals directly affect the presynaptic calcium current.

However, it was later shown that subthreshold analogue signals propagate several hundreds of micrometers along an axon and modulate action potential-evoked transmitter release at the synapses (Alle and Geiger, 2006; Shu et al., 2006). This may enhance the computational abilities of neurons (Figure 1A right). However, the mechanisms mediating analogue-digital signaling in axons remain controversial.

Previous studies have suggested that analogue-digital signaling can modify neurotransmitter release in three ways (Figure 1B; for an extensive review and other potential mechanisms see Zbili and Debanne, 2019). First, analogue signals with a positive potential can increase the duration of the action potentials by inactivating voltage-dependent potassium channels. Second, positive analogue signals can increase the amplitude of the action potentials by keeping voltage-dependent sodium channels active under certain conditions. A longer and larger action potential opens voltage-dependent calcium channels more efficiently and thereby increases neurotransmitter release. Third, positive analogue signals can also increase the resting calcium concentration inside the synapse, leading to a higher probability that neurotransmitter will be released.

Now, in eLife, Federico Trigo (Instituto de Investigaciones Biológicas Clemente Estable, Uruguay) and Shin-ya Kawaguchi (Kyoto University, Japan) report on a new mechanism explaining analogue-digital signaling in the brain (Trigo and Kawaguchi, 2023). The researchers combined their expertise in sub-cellular patch-clamp recordings to analyze the mechanisms of analogue-digital signaling in both acutely prepared brain slices of rats and in neurons grown in cell culture. This enabled them to simultaneously record signals from the cell body of the neuron and from the axon terminals of a specific type of neuron, known as cerebellar molecular layer interneuron.

During the presynaptic patch-clamp recordings, the researchers applied recorded action potentials as voltage commands. They discovered that weak and short positive potentials (resembling the analogue signals of synapses) before an action potential increased both calcium influx into the axon and the release of neurotransmitters. This was surprising since the waveform of the presynaptic action potential was identical. Therefore, the first two mechanisms described above (which involved changes in the duration or amplitude of the action potential) cannot explain the results.

To address if the third mechanism involving an elevated resting calcium concentration can explain their results, Trigo and Kawaguchi interrupted the positive potentials before the action potentials for only a few milliseconds. Such a short interruption should not affect the resting calcium concentration. Yet, the analogue modulation was abolished, indicating that the third mechanism cannot explain the results either.

With the help of computer models, the researchers could demonstrate that analogue signals directly modulate calcium channels by increasing their readiness to open. This transition depends on voltage but is not mediated by the calcium-dependent modulation of calcium channels (Lin et al., 2012). In other words, the analogue signal switches the channels into a primed state, which facilitates opening once the action potential arrives. The calcium channels thus ‘remember’ the analogue signal until the action potential invades the synapse. However, when analogue signals are briefly interrupted, the channels rapidly revert to the resting conformation state, which prevents the analogue modulation of the calcium channels. A similar mechanism has previously been found in presynaptic potassium channels (Yang et al., 2014).

The study by Trigo and Kawaguchi raises several interesting points. Their recordings were mainly performed in immature neurons, but the properties of presynapses change profoundly as neurons mature (Taschenberger and von Gersdorff, 2000). It remains to be seen if analogue-digital signaling changes during maturation. Synapses are also extremely diverse, and more research is needed to see if the new mechanism is a specialized feature of interneurons in the cerebellar circuitry or would also apply to other neurons (Nusser, 2018). Interestingly, some synapses in the auditory pathway have remarkable stable presynaptic action potentials in vivo, which would allow analogue signaling to be mediated by intermediate gating states of calcium channels (Sierksma and Borst, 2017). Moreover, axons can sometimes emerge from dendrites, and it is unknown if analogue-digital signaling would be increased in these cases (Kole and Brette, 2018). Finally, intermediate gating states could be an important modulation of the complete failure of action potential propagation (Kawaguchi and Sakaba, 2015).

In summary, Trigo and Kawaguchi have performed a series of technically challenging experiments that revealed a new mechanism of analogue-digital signal transmission in the axon. This demonstrated that axons are more than simple digital conduction devices. Instead, past analogue signals in the axon modulate synaptic output to which the intermediate gating states of calcium channels contribute.
